# ZBTB7A mutations in acute myeloid leukaemia with t(8;21) translocation

**DOI:** 10.1038/ncomms11733

**Published:** 2016-06-02

**Authors:** Luise Hartmann, Sayantanee Dutta, Sabrina Opatz, Sebastian Vosberg, Katrin Reiter, Georg Leubolt, Klaus H. Metzeler, Tobias Herold, Stefanos A. Bamopoulos, Kathrin Bräundl, Evelyn Zellmeier, Bianka Ksienzyk, Nikola P. Konstandin, Stephanie Schneider, Karl-Peter Hopfner, Alexander Graf, Stefan Krebs, Helmut Blum, Jan Moritz Middeke, Friedrich Stölzel, Christian Thiede, Stephan Wolf, Stefan K. Bohlander, Caroline Preiss, Linping Chen-Wichmann, Christian Wichmann, Maria Cristina Sauerland, Thomas Büchner, Wolfgang E. Berdel, Bernhard J. Wörmann, Jan Braess, Wolfgang Hiddemann, Karsten Spiekermann, Philipp A. Greif

**Affiliations:** 1 Department of Internal Medicine 3, University Hospital, Ludwig-Maximilians-Universität (LMU) München, 81377 München, Germany; 2 Clinical Cooperative Group Leukemia, Helmholtz Zentrum München, German Research Center for Environmental Health, 81377 München, Germany; 3 German Cancer Consortium (DKTK), 69121 Heidelberg, Germany; 4 German Cancer Research Center (DKFZ), 69121 Heidelberg, Germany; 5 Department of Biochemistry, Ludwig-Maximilians-Universität (LMU) München, 81377 München, Germany; 6 Laboratory for Functional Genome Analysis (LAFUGA), Gene Center, Ludwig-Maximilians-Universität (LMU) München, 81377 München, Germany; 7 Medizinische Klinik und Poliklinik I, Universitätsklinikum Dresden, 01307 Dresden, Germany; 8 Department of Molecular Medicine and Pathology, The University of Auckland, Auckland 1142, New Zealand; 9 Department of Transfusion Medicine, Cell Therapeutics and Hemostasis, University Hospital, Ludwig-Maximilians-Universität (LMU) München, 81377 München, Germany; 10 Institute of Biostatistics and Clinical Research, University of Münster, 48149 Münster, Germany; 11 Department of Medicine A, Hematology, Oncology and Pneumology, University of Münster, 48149 Münster, Germany; 12 Department of Hematology, Oncology and Tumor Immunology, Charité University Medicine, Campus Virchow, 13353 Berlin, Germany; 13 Oncology and Hematology, St. John-of-God Hospital, 93049 Regensburg, Germany

## Abstract

The t(8;21) translocation is one of the most frequent cytogenetic abnormalities in acute myeloid leukaemia (AML) and results in the *RUNX1*/*RUNX1T1* rearrangement. Despite the causative role of the *RUNX1*/*RUNX1T1* fusion gene in leukaemia initiation, additional genetic lesions are required for disease development. Here we identify recurring *ZBTB7A* mutations in 23% (13/56) of AML t(8;21) patients, including missense and truncating mutations resulting in alteration or loss of the C-terminal zinc-finger domain of ZBTB7A. The transcription factor ZBTB7A is important for haematopoietic lineage fate decisions and for regulation of glycolysis. On a functional level, we show that *ZBTB7A* mutations disrupt the transcriptional repressor potential and the anti-proliferative effect of ZBTB7A. The specific association of *ZBTB7A* mutations with t(8;21) rearranged AML points towards leukaemogenic cooperativity between mutant ZBTB7A and the RUNX1*/*RUNX1T1 fusion.

## Abstract (web summary)

The t(8;21) translocation is often found in acute myeloid leukaemia but is not sufficient for development of the disease. In this study, the authors identify frequent mutations in the transcriptional repressor, *ZBTB7A*, in these patients and show that the mutations reduce DNA binding activity.

## Graphical abstract

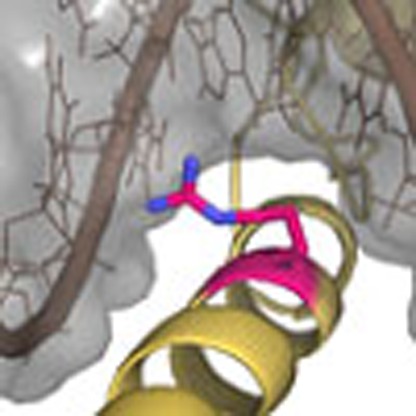

Block of myeloid differentiation is one of the hallmarks of acute myeloid leukaemia (AML). First insights into this key mechanism were gained by the discovery of the t(8;21)(q22;q22) translocation, which was the first balanced translocation described in a tumour and results in the *RUNX1*/*RUNX1T1* fusion gene (also known as AML1/ETO)^1^,^2^. The *RUNX1*/*RUNX1T1* rearrangement is one of the most frequent chromosomal aberrations in AML and defines an important clinical entity with favourable prognosis according to the World Health Organization classification^3^. The RUNX1/RUNX1T1 fusion protein disrupts the core-binding factor complex, and thereby blocks myeloid differentiation. However, *in vivo* models indicate the requirement of additional lesions, such as of *KIT* or *FLT3* mutations, for leukaemogenesis as the *RUNX1*/*RUNX1T1* fusion gene alone is not sufficient to induce leukaemia^4^,^5^,^6^,^7^,^8^. In the present study, we set out to identify additional mutations in AML t(8;21) and discovered frequent mutations of *ZBTB7A*—encoding a transcription factor important for the regulation of haematopoietic development^9^ and tumour metabolism^10^. It is very likely that *ZBTB7A* mutations are one of the important missing links in *RUNX1*/*RUNX1T1*-driven leukaemogenesis.

## Results

### *ZBTB7A* is frequently mutated in AML t(8;21)

**Figure 1:**
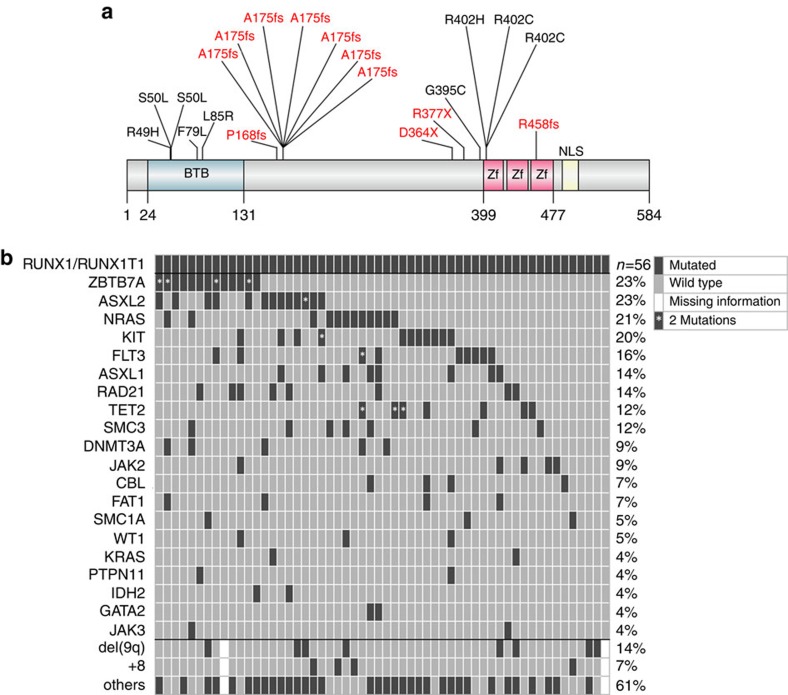
*ZBTB7A* mutations in AML t(8;21). (**a**) ZBTB7A protein (NP_056982.1) and identified mutations (red=truncating; black=missense) illustrated using IBS software^31^. Amino-acid positions are indicated below the graph. BTB, BR-C ttk and bab; NLS, nuclear localization sequence; Zf, zinc finger. (**b**) Mutational landscape of 56 diagnostic AML samples with t(8;21) translocation. Each column represents one patient, each line one of the analysed genes or cytogenetic markers.

**Figure 2:**
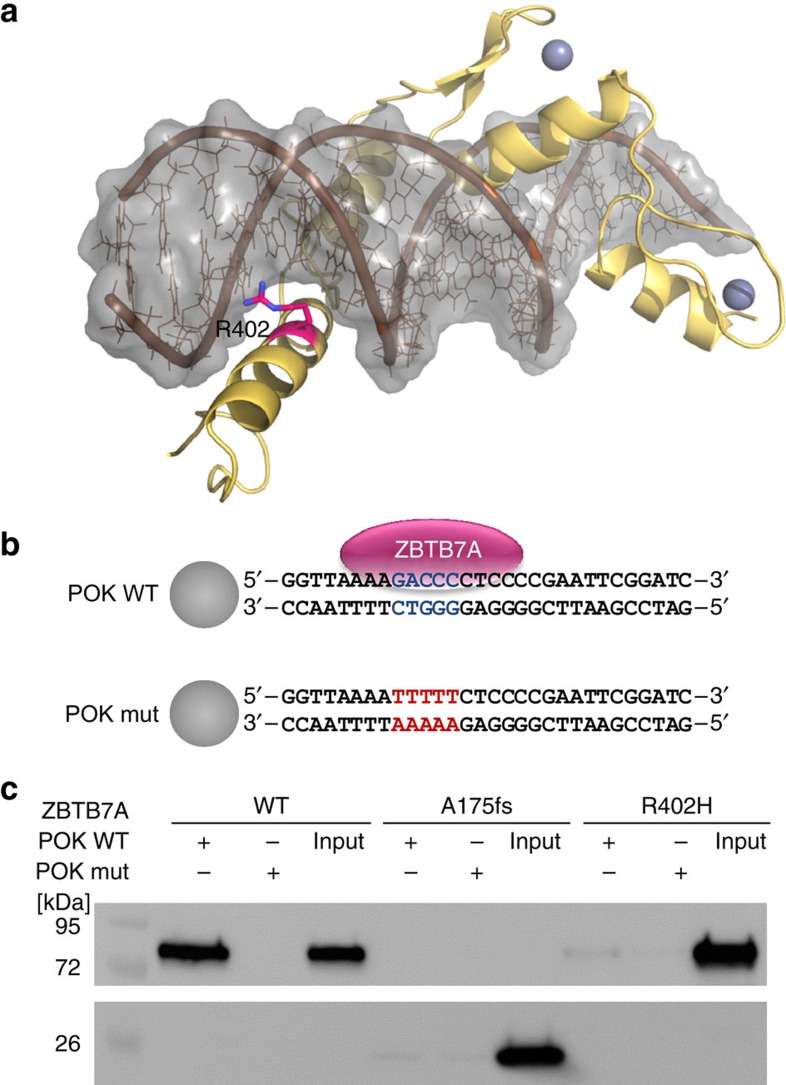
Impact of ZBTB7A mutations on DNA binding. (**a**) Model for the C-terminal zinc-finger domain of ZBTB7A comprising residues 382–488. The model is depicted as yellow ribbon with highlighted secondary structure. Zinc ions are shown as grey spheres. DNA is shown in brown with a grey molecular surface. R402 (purple) binds into the major groove and likely contributes to the affinity or sequence specificity of the DNA interaction of the zinc-finger domain. (**b**) Biotinylated oligonucleotides containing the ZBTB7A (alias: Pokemon) consensus binding motif (POK WT) or a mutant thereof (POK mut)^14^ used in DNA pull-down experiments. Spheres illustrate streptavidin-coated beads. (**c**) DNA pull-down using protein lysates from HEK293T cells expressing wild-type or mutant ZBTB7A. Western blot analysis shows that A175fs and R402H fail to bind oligonutides with a ZBTB7A-binding site (POK WT). Oligonucleotides with a mutated binding site (POK mut) were used as negative control. Input lanes were loaded with 10% of the protein lysate used for each binding reaction.

To identify additional cooperating mutations, we performed exome sequencing of matched diagnostic and remission samples from two AML patients with t(8;21) translocation and detected 11 and 12 somatic variants, respectively (Supplementary Table 1). *ZBTB7A* was the only mutated gene identified in both patients. ZBTB7A (also known as LRF, Pokemon and FBI-1) is a member of the POZ/BTB and Krüppel (POK) transcription factor family^9^, which is characterized by an N-terminal POZ/BTB protein–protein interaction domain and C-terminal C_2_H_2_ zinc fingers^11^. The first patient carried a homozygous missense mutation resulting in the amino-acid change R402H (NM_015898:exon2:c.1205G>A:p.R402H) affecting the highly conserved zinc-finger domain, while a heterozygous frameshift insertion (NM_015898:exon2:c.522dupC:p.A175fs) resulting in loss of the zinc-finger domain was identified in the second patient. Both mutations were validated by Sanger sequencing (Supplementary Fig. 1; Supplementary Table 2). Using targeted amplicon sequencing of *ZBTB7A* and 45 leukaemia relevant genes, we screened 56 diagnostic AML t(8;21) samples, including one of the two samples analysed by exome sequencing (UPN 1), whereas for the other one (UPN 2) availability of material was insufficient. *ZBTB7A* mutations were identified in 13 of 56 patients (23%; Fig. 1a,b; Supplementary Table 3). Patient characteristics are summarized in Supplementary Table 4. Two recurring mutational hotspots (A175fs and R402) in exon 2 were identified altering or resulting in loss of the zinc-finger domain (Fig. 1a). It was previously shown that the zinc-finger domain of *ZBTB7A* is essential for DNA binding^12^. Structural modelling revealed that arginine 402 binds into the major groove of the DNA double helix and likely contributes to the affinity or sequence specificity of the DNA interaction of the zinc-finger domain of ZBTB7A (Fig. 2a). We confirmed that both ZBTB7A mutants A175fs and R402H fail to bind DNA (Fig. 2b,c).

Variant allele frequency ranged from 5.4 to 76.2% (cut-off 2%) and 4 of 13 patients (31%) harboured two mutations of *ZBTB7A*. Fourteen of 17 mutations (82%) were validated by Sanger sequencing (Supplementary Fig. 1). Somatic status was confirmed in a total of three patients with available remission samples. Thirty-two additional samples of t(8;21)-positive AML with inadequate sample availability for gene panel sequencing were analysed by Sanger sequencing of exon 2 (encoding amino acids 1–421) resulting in the identification of two *ZBTB7A* mutations (2/32; 6%). This lower mutation frequency might be due to the lower sensitivity of Sanger sequencing and incomplete coverage of the coding exons of *ZBTB7A* (we were not able to reliably amplify exon 3 encoding amino acids 422–584). To evaluate the consequences of truncating *ZBTB7A* mutations on the protein level, we performed western blot analysis for one patient with available material and detected a shorter form of the ZBTB7A protein resulting from the R377X mutation (Supplementary Fig. 2).

Recently, frequent *ASXL2* mutations were identified in t(8;21) AML^13^. In our cohort, *ZBTB7A* and *ASXL2* mutations occurred at similar frequencies (Fig. 1b) and 5 of 13 patients carried mutations in both genes; however, there was no significant association of mutated *ZBTB7A* and mutations in *ASXL2* (Fisher's exact test, *P*=0.12) or any other recurrently mutated gene. Alterations of *ASXL1* were mutually exclusive with genetic lesions of *ZBTB7A* suggesting alternative routes of leukaemogenesis. Similarly, mutations of *ZBTB7A* and *KIT* were exclusive in all, but one patient. In the exome data of 22 patients with inversion inv(16) (another rearrangement disrupting the core-binding factor complex in AML), we found a single *ZBTB7A* mutation (A211V). Of note, we did not find any *ZBTB7A* mutations by exome sequencing of 50 patients with cytogenetically normal AML (CN-AML) or 14 AML patients with chromosomal aberrations other than t(8;21) or inv(16). These results point towards a specific association between *ZBTB7A* alterations and the *RUNX1*/*RUNX1T1* fusion.

### Mutations disrupt the anti-proliferative function of ZBTB7A

**Figure 3:**
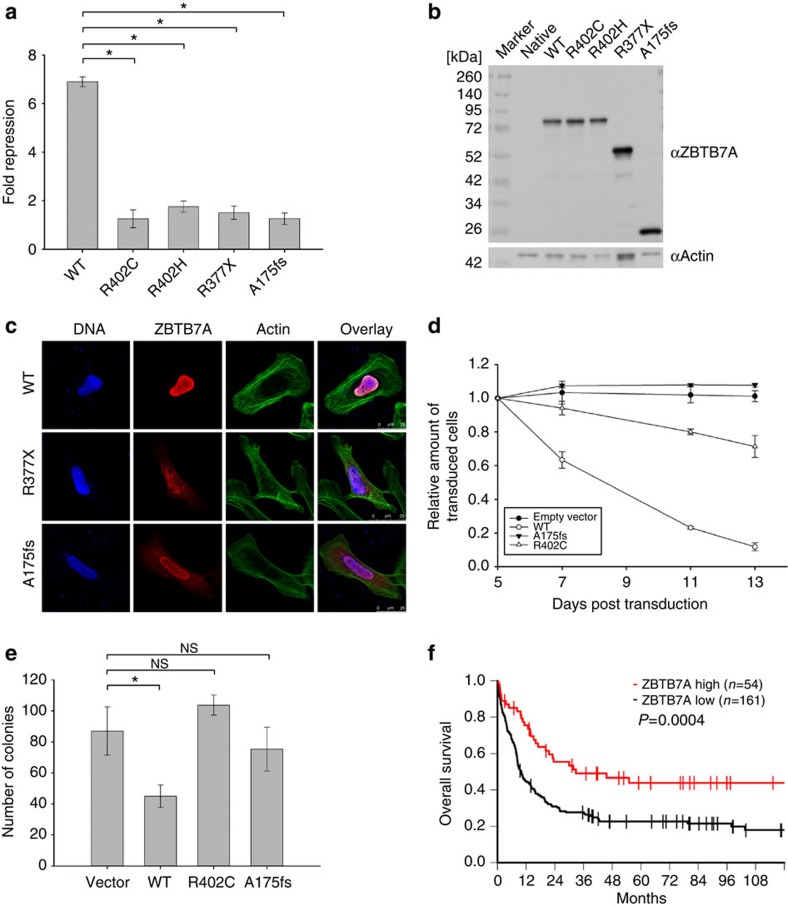
Functional consequences of *ZBTB7A* mutations and clinical relevance of *ZBTB7A* expression. (**a**) Luciferase assay in transiently transfected HEK293T cells using the pGL2-p19ARF-Luc reporter combined with expression constructs for wild-type and mutant ZBTB7A. (**b**) Western blot of ZBTB7A constructs expressed in HEK293T cells. (**c**) Sub-cellular localization of ZBTB7A wild type, R377X and A175fs in transiently transfected U2OS cells. Scale bar, 25 μm. (**d**) Growth of Kasumi-1 cells stably expressing ZBTB7A wild type or mutants. (**e**) CFC assay of murine bone marrow lineage-negative cells co-expressing RUNX1/RUNX1T1 and wild-type or mutant ZBTB7A. (**f**) Overall survival of patients with CN-AML according to *ZBTB7A* expression (log-rank test, *P*=0.0004). *Two-tailed, unpaired Student's *t*-test, *P*<0.05; NS, not significant. Bar graphs or growth curves represent mean±s.d. of three independent experiments.

To assess the functional consequences of the identified *ZBTB7A* mutations, we performed luciferase reporter gene assays. It is known that ZBTB7A represses the expression of *ARF* (alternate open reading frame of *CDKN2A*)^14^. In contrast to wild-type ZBTB7A, the R402H, R402C, A175fs or R377X mutants failed to repress a luciferase reporter containing ZBTB7A-binding elements derived from the *ARF* promoter (Fig. 3a). Expression of ZBTB7A constructs was confirmed by western blot (Fig. 3b).

In light of recent reports about the negative regulation of glycolysis by ZBTB7A^10^, we assessed the expression of glycolytic genes (*SLC2A3*, *PFKP* and *PKM*) in the RNA-sequencing data from our AML t(8;21) patients (Supplementary Fig. 3). In *ZBTB7A*-mutated patients (*n*=5), we found a significantly higher expression of *PFKP* (Student's *t*-test, *P*=0.03) compared with patients without any detectable *ZBTB7A* mutation (*n*=11). On average, *PKM* and *SLC2A3* also showed higher expression levels in patients with *ZBTB7A* mutations, but did not reach statistical significance (Student's *t*-test, *P*=0.17 and *P*=0.54, respectively). In the latter case, the difference in the mean values can be attributed mainly to an outlier in the *ZBTB7A*-mutated group with very high *SLC2A3* expression. Expression levels of *ZBTB7A* were similar in both the patient groups, compatible with inactivation of *ZBTB7A* on the genetic level rather than on the transcriptional level.

The C-terminal part of ZBTB7A is important for nuclear localization^15^. Because some mutations result in loss of the C-terminal zinc-finger domain and nuclear localization signal, we evaluated the cellular localization of mutant ZBTB7A. Whereas wild-type ZBTB7A was detected in the nucleus, immunofluorescence staining of the A175fs and R377X mutants showed an altered cytoplasmic localization (Fig. 3c). In contrast, mutants R402H and R402C exhibited a variable cellular localization with cytoplasmic protein detectable only in a minor subset of cells (Supplementary Fig. 4a,b). Amino-acid substitutions of R402 showed a smaller increase in cytoplasmic protein fraction compared with truncation mutants as analysed by western blot (Supplementary Fig. 4c). Ultimately, the observed effect of mutations on ZBTB7A localization remains to be confirmed in appropriate primary patient material, which was not available in our study.

In the t(8;21) translocation-positive AML cell line Kasumi-1, retroviral expression of wild-type *ZBTB7A* inhibited cell growth, whereas this anti-proliferative effect was not observed upon expression of the A175fs *ZBTB7A* mutant (Fig. 3d). The R402C mutant expressing Kasumi-1 cells showed a trend towards reduced cell growth, suggesting residual activity. On the basis of this observation, we expressed *ZBTB7A* wild type or its mutants together with the *RUNX1*/*RUNX1T1* fusion in lineage-negative murine bone marrow cells and performed colony-forming cell (CFC) assays. *ZBTB7A* expression led to a significant decrease in the number of colonies in primary CFC (87±12.6 versus 45±5.8, Student's *t*-test, *P*<0.0001), while this effect was lost for both mutants tested (Fig. 3e). These findings support an oncogenic cooperativity between *RUNX1*/*RUNX1T1* and *ZBTB7A* mutations.

### Prognostic relevance of *ZBTB7A* expression in CN-AML

The identification of a novel recurrently mutated gene demands the evaluation of its clinical relevance. We did not find a significant difference in overall or relapse-free survival between t(8;21)-positive AML patients with wild-type or mutant *ZBTB7A* (Supplementary Fig. 5). However, this evaluation was limited by the relatively small cohort size. Considering the potential role of *ZBTB7A* as tumour suppressor in AML and its anti-proliferative properties, we correlated *ZBTB7A* expression with clinical outcome in a larger cohort of AML patients (GSE37642). There was no significant difference in *ZBTB7A* expression levels between cytogenetic subgroups of AML (Supplementary Fig. 6). Remarkably, in over 200 CN-AML patients treated on clinical trial (NCT00266136), high expression of *ZBTB7A* was associated with a favourable outcome (Fig. 3f; Supplementary Fig. 7), suggesting a relevance in AML beyond the t(8;21) subgroup. The favourable prognostic impact of high *ZBTB7A* transcript levels was most obvious in elderly patients (age >60 years) and high *ZBTB7A* expression was associated with a ‘low molecular risk genotype' (mutated *NPM1* without *FLT3*-ITD; Supplementary Fig. 7; Supplementary Table 5). We validated the association of high *ZBTB7A* expression with favourable outcome in an independent CN-AML patient cohort^16^,^17^ (Supplementary Fig. 8).

## Discussion

In summary, we have identified *ZBTB7A* as one of the most frequently mutated genes in t(8;21)-positive AML. Consistent with our findings, *ZBTB7A* mutations in 3 of 20 (15%) AML t(8;21) patients and 1 of 395 AML inv(16) patients were reported^18^ during the revision of the present manuscript. Our functional analyses indicate that *ZBTB7A* mutations result in loss of function, due to alteration or loss of the zinc-finger motives. Beyond DNA binding, the zinc-finger domain of ZBTB7A is also known to interact with TP53 and BCL6 (ref. 9). Thus, multiple pathways might be influenced by alteration or loss of the ZBTB7A zinc-finger domain. The N-terminal missense mutations in the BTB domain may result in failure of co-repressor recruitment. Considering that 4 of 13 of patients had more than one *ZBTB7A* mutation, our finding that overexpression of wild-type *ZBTB7A* leads to reduced proliferation of Kasumi-1 cells and a decreased number of CFCs of murine bone marrow cells, we suggest that *ZBTB7A* acts as a tumour suppressor in t(8;21)-positive AML. Initial studies characterized *ZBTB7A* as proto-oncogene in various tissues^14^,^19^. For example, Maeda *et al*. demonstrated that transgenic mice with *Zbtb7a* overexpression in the immature T- and B-lymphoid lineage develop precursor T-cell lymphoma/leukaemia^14^. In contrast, it was more recently shown that *ZBTB7A* can also act as a tumour suppressor. Overexpression of *Zbtb*7a in murine prostate epithelium did not result in neoplastic transformation; unexpectedly, *Zbtb7a* inactivation lead to the acceleration of Pten-driven prostate tumorigenesis^20^. Recently, somatic zinc-finger mutations of *ZBTB7A* were found at low frequencies (<5%) in a variety of solid cancers suggesting a common mechanism across tumour entities^21^. In fact, the de-repression of glycolytic genes upon deletion or mutation of *ZBTB7A*^10^,^21^ might underlie the loss of anti-proliferative properties that we observed for ZBTB7A mutants A175fs and R402C in the present study. Any inactivating alteration of *ZBTB7A* will likely increase glycolysis, and, thus, helps the tumour cells to produce more energy. Besides tumour metabolism, it is known that ZBTB7A also plays an important role in haematopoietic lineage fate decisions^9^. During lymphopoiesis *ZBTB7A* regulates B-cell development^22^, whereas in the myeloid lineage it is essential for erythroid differentiation^23^. Thus, *ZBTB7A* mutations may contribute to the block of differentiation in AML t(8;21).

The favourable prognostic relevance of high *ZBTB7A* expression in CN-AML, which accounts for half of all AML patients, may point towards a more general tumour suppressor role of *ZBTB7A* in myeloid leukaemia. In particular, the anti-proliferative properties of ZBTB7A may slow down disease progression. High *ZBTB7A* expression as a favourable prognostic marker has been reported also in colorectal cancer^10^, consistent with a clinicobiological role of *ZBTB7A* across malignancies of multiple tissue origins. Given that somatic mutations of *ZBTB7A* seem to be absent or rare in CN-AML, other mechanisms, including epigenetic changes or alterations of upstream regulators, may lead to inactivation or downregulation of *ZBTB7A.*

Our discovery of frequent *ZBTB7A* mutations in AML with t(8;21) translocation, one of the most common translocations in AML and the first balanced translocation identified in leukaemia^1^, demonstrates that the mutational landscape of AML is still not fully understood. Further studies will be required to unravel the mechanism underlying leukaemogenic cooperativity between mutated *ZBTB7A* and the *RUNX1*/*RUNX1T1* fusion gene.

## Methods

### Patients

AML samples were collected within the German Cancer Consortium (DKTK) at the partner sites Munich and Dresden. Patients were treated according to the protocols of Acute Myeloid Leukemia Cooperative Group (AMLCG) or Study Alliance Leukemia (SAL) multicentre clinical trials. Study protocols were approved by the Institutional Review Boards of the participating centres. Informed consent was received in accordance with the Declaration of Helsinki.

### Sequencing

Exome sequencing (mean coverage: 87x; range 80–90x) was performed on a HiSeq 2000 Instrument (Illumina), using the SureSelect Human All Exon V5 kit (Agilent). Pretreatment blood or bone marrow specimens from 56 AML patients with t(8;21) translocation were sequenced using Haloplex custom amplicons (Agilent) and a HiSeq 1500 instrument (Illumina). Target sequence included the entire open-reading frame of *ZBTB7A* in addition to 45 leukaemia-related genes or mutational hotspots (Supplementary Table 3). Variant calling was performed as described previously^24^. Sanger sequencing of PCR-amplified genomic DNA was carried out using a 3500xL Genetic Analyzer (Applied Biosystems). Primer sequences are provided in Supplementary Table 2. Sequencing of messenger RNA was performed using the TruSeq RNA Sample Preparation protocol, followed by sequencing on a HiSeq 2000 Instrument (Illumina). RNA sequence reads were aligned to the human genome (hg19) using STAR^25^ (version 2.4.1b). Reads per gene were counted using HTseq^26^ (version 0.6.1) with intersection-strict mode and normalized for the total number of reads per sample.

### Structural modelling

Suitable templates for the modelling were searched with HHPRED^27^, using the zinc-finger domain of ZBTB7A as input sequence. The highest scoring homologue, for which a structure of a DNA complex is available, was the Wilms tumour suppressor protein^28^ (PDB accession code 2J9P, *E*-value 4.8E–29, *P*-value 1.3E–30). The model for ZBTB7A was generated on the basis of 2J9P using MODELLER^29^. Importantly, 2J9P also contains an arginine at the equivalent position of ZBTB7A's R402, allowing us to model the function of R402 as major groove binder with confidence.

### Plasmids

The pcDNA3.1-His-ZBTB7A expression construct was a gift from Takahiro Maeda (Boston). ZBTB7A A175fs, R377X, R402C and R402H mutant plasmids were generated using the QuikChange II XL Site-Directed Mutagenesis Kit (Agilent) and confirmed by Sanger sequencing. *ZBTB7A* wild type and mutants were subcloned into pMSCV-IRES-YFP (pMIY), using the In-Fusion HD cloning kit (Clontech) and EcoRI restriction sites. The pMSCV-IRES-GFP(pMIG)-RUNX1/RUNX1T1 plasmid was provided by Christian Buske (Ulm).

### DNA pull-down

HEK293T cells (DSMZ no.: ACC 635) were transfected with pcDNA3.1 His-Xpress-ZBTB7A (wild type or mutant). After 24 h, protein was extracted using lysis buffer (50 mM Tris HCl, pH 8.5, 150 mM NaCl, 1% Triton X-100, cOmplete Protease Inhibitor Cocktail). For each reaction, 20 μl protein lysate was incubated in binding buffer (PBS supplemented with 150 mM NaCl resulting in a total salt concentration of nearly 300 mM, 0.1% NP40, 1 mM ETDA) with 10 pM biotinylated double-stranded oligonucleotides that contain either the ZBTB7A consensus binding motif (POK WT; 5′-GGTTAAAAGACCCCTCCCCGAATTCGGATC-3′) or a mutant thereof (POK mut; 5′-GGTTAAAATTTTTCTCCCCGAATTCGGATC-3′). After 1 h of incubation at 4 °C, 10 μl streptavidin agarose beads (Sigma Aldrich) was added to each reaction and incubated for 30 min at 4 °C. Beads were washed three times with binding buffer and resupended in 10 μl Laemmli buffer for subsequent western blot analysis. ZBTB7A protein was detected using an antibody against the Xpress tag (1:5,000 dilution, clone R910-25; Life Technologies) and secondary goat anti-mouse IgG-HRP (1:10,000 dilution, clone sc-2060; Santa Cruz). The uncropped western blot scan underlying Fig. 2c is shown in Supplementary Fig. 9.

### Reporter gene assay

HEK293T cells (DSMZ no.: ACC 635) were co-transfected with pcDNA3.1-His-ZBTB7A (wild type or mutant), pGL2-p19ARF-Luc (gift from Takahiro Maeda, Boston) as well as pRL-CMV (Renilla luciferase; Promega) using Lipofectamine 2000 (ThermoFischer). After 24 h, cells were lysed; Firefly and Renilla luciferase activity was measured with the dual-luciferase reporter assay system (Promega) according to the manufacturer's instructions. Three independent experiments were each performed in triplicates.

### Western blot

HEK293T cells (DSMZ no.: ACC 635) were transfected using Lipofectamine 2000 (ThermoFischer) with pcDNA3.1-His-ZBTB7A (wild type or mutant). After 24 h, protein was either extracted by multiple freeze–thaw cycles in lysis buffer (600 mM KCl, 20 mM Tris-Cl pH 7.8, 20% Glycerol, cOmplete Protease Inhibitor Cocktail) or using the Qproteome Nuclear Protein Kit (Qiagen) for the analysis of nuclear and cytoplasmic protein fractions. From archived patient bone marrow samples, protein was isolated using the AllPrep DNA/RNA/Protein Mini Kit (Qiagen) according to the manufacturer's instructions. Following SDS–polyacrylamide gel electrophoresis and protein transfer to polyvinylidene difluoride membrane (Hybond PTM, Amersham Pharmacia biotech), immunoblots were blocked with 5% nonfat dried milk, probed with anti-human Pokemon (ZBTB7A) purified antibody (1:5,000 dilution, clone: 13E9; eBioscience) and secondary anti-Armenian hamster IgG-HRP (1:10,000 dilution, clone: sc-2443; Santa Cruz). As loading control immunoblots were incubated with rabbit anti-actin (1:5,000 dilution, clone: sc-1616- R; Santa Cruz) and secondary goat anti-rabbit IgG-HRP (1:10,000 dilution, clone: sc-2030; Santa Cruz). For analysis of the nuclear and cytoplasmic ZBTB7A protein fractions, we used mouse anti-Xpress tag (1:5,000 dilution, clone R910-25, Life Technologies) and secondary goat anti-mouse IgG-HRP (1:10,000 dilution, clone: sc-2060; Santa Cruz). Mouse anti-GAPDH (1:10,000 dilution, clone: sc-32233; Santa Cruz) served as loading control for the cytoplasmic protein fraction. Proteins were detected with enhanced chemiluminescence (ECL, Amersham, GE Healthcare).

### Immunofluorescence staining

U2OS human osteosarcoma cells (ATTC no.: HTB-96) were grown on coverslips and transiently transfected with pcDNA3.1-His-ZBTB7A wild type and mutant constructs using PoliFect (Qiagen) according to the manufacturer's guidelines. Cells were fixed 48 h post transfection using PBS 2% formaldehyde (37% stock solution; Merck Schuchardt) for 10 min, permeabilized with PBS 0.5% Triton X-100 (Carl Roth) for 10 min and blocked for 1 h with PBS 2% bovine serum albumin (Albumin Fraction V, AppliChem). Cells were then incubated with polyclonal rabbit His-probe (H-15) antibody (1:50 dilution; Santa Cruz) for 1 h. After extensive washing with PBS 0.1% Tween 20 (Carl Roth), secondary antibody incubation was performed for 1 h with goat anti-rabbit IgG (H+L), F(ab′)2 fragment Alexa Fluor 594 conjugate (1:500 dilution; Cell Signaling Technology). Counterstaining was performed using NucBlue Reagent and ActinGreen 488 ReadyProbes Reagent (Life Technologies; 2 drops per ml) at room temperature for 20 min. Coverslips were mounted using fluorescence mounting medium (DAKO). Specimens were analysed using a confocal fluorescence laser scanning system (TCS SP5 II; Leica). For image acquisition and processing, the LAS AF Lite Software (Leica) was used.

### Retroviral transduction

Retroviral transduction of Kasumi-1 cells (DSMZ no.: ACC 220) was accomplished as outlined previously^30^. In brief, HEK293T cells were co-transfected with pMSCV-IRES-YFP (pMIY) vectors containing either wild-type or mutant (A175fs, R402C) ZBTB7A and packaging plasmids. After 48 h, the cell culture supernatant was collected, sterile filtered and used for viral loading of RetroNectin (Takara Clontech)-coated plates. A total of 3 × 10^5^ Kasumi-1 cells were transduced per well. The percentage of YFP-positive cells was assessed on a FACSCalibur flow cytometer (BD Biosciences). Three independent experiments were each performed in duplicates.

### Colony-forming cell assay

For *in vitro* CFC assays, bone marrow cells were collected from the femur and pelvic girdle of wild-type mice (C57BL/6X129/J). Lineage-negative haematopoietic progenitors were isolated using magnetic separation (MACS, murine lineage depletion kit, Miltenyi biotech). Retrovirally transduced cells were sorted for GFP/YFP and were plated in 1% myeloid-conditioned methylcellulose containing Iscove's modified Dulbecco medium-based Methocult (Methocult M3434; StemCell Technologies) at a concentration of 500 cells per ml. Single-cell suspensions of colonies were serially replated at the same concentration until the exhaustion of cell growth. Three independent experiments were each performed in duplicates.

### Analysis of clinical and gene expression data

Clinical relevance of *ZBTB7A* mutations or expression levels was evaluated using the Kaplan–Meier method and the log-rank test. Fisher's exact test was used to compare categorical variables, while Wilcoxon Mann–Whitney *U*-test was applied for continuous variables. All patients included in this analysis were treated intensively with curative intent according to the AMLCG protocols. Gene expression profiling was performed on 215 adult patients with cytogenetically normal AML, using Affymetrix Human Genome (HG) U133A/B (*n*=155) and HG U133Plus2.0 microarrays (*n*=60). The RMA method was used for data normalization, and probe set summarization utilized custom chip definition files based on the GeneAnnot database (version 2.2.0). Probe set GC19M004001_at was used to determine *ZBTB7A* expression levels. High *ZBTB7A* expression was defined as the highest (4th) quartile of expression values observed in CN-AML patients. Patients with *ZBTB7A* expression levels in the 1st to 3rd quartile were classified as having low expression. The patients analysed here represent a subset of the previously published data set GSE37642. Validation of the results was done using data sets from the Haemato Oncology Foundation for Adults in the Netherlands (HOVON) study group (GSE14468 and GSE1159)^16^,^17^.

### Data availability

Data referenced in this study are available in the Gene Expression Omnibus database with the accession codes GSE37642, GSE14468 and GSE1159. The next-generation sequencing data that support the findings of this study are available on request from the corresponding author (P.A.G). The data are not publicly available due to them containing information that could compromise research participant privacy or consent. Explicit consent to deposit raw-sequencing data was not obtained from the patients, many samples were collected >10 years ago. Thus, the vast majority of patients cannot be asked to provide their consent for deposit of their comprehensive genetic data.

## Additional information

**How to cite this article:** Hartmann, L. *et al*. ZBTB7A mutations in acute myeloid leukaemia with t(8;21) translocation. *Nat. Commun.* 7:11733 doi: 10.1038/ncomms11733 (2016).

## Supplementary Material

Supplementary Information Supplementary Figures 1-9 and Supplementary Tables 1-5.
