# Supplementary material for: ZBTB7A mutations in acute myeloid leukaemia with t(8;21) translocation
Source: Nat Commun. 2016 Jun 2;7:11733. doi: 10.1038/ncomms11733 (PMC4895769; doi:10.1038/ncomms11733)
Supplement: Supplementary Information — Supplementary Figures 1-9 and Supplementary Tables 1-5. [file ncomms11733-s1.pdf]

UPN#1-Diagnosis: NM_015898:exon2: c.1205G>A:p.R402H

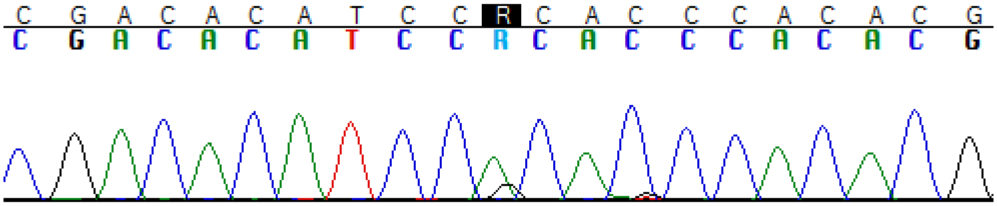

UPN#1-Remission: Wild-type

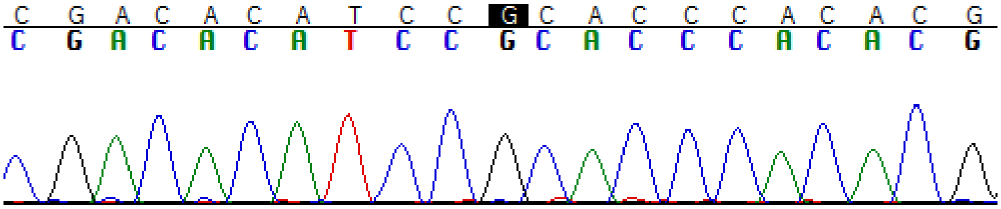

UPN#2-Diagnosis: NM_015898:exon2: c.522dupC:p.A175fs

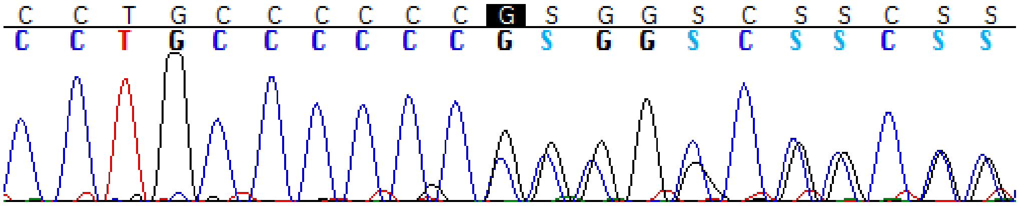

UPN#2-Remission: Wild-type

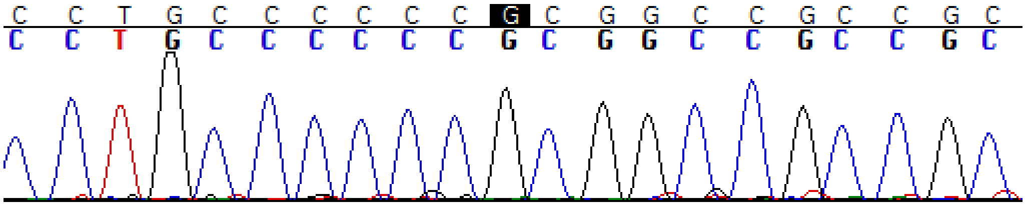

Supplementary Figure 1. Sanger sequencing confirms *ZBTB7A* mutations.

UPN#3-Diagnosis: NM_015898:exon2: c.1204C>T:p.R402C

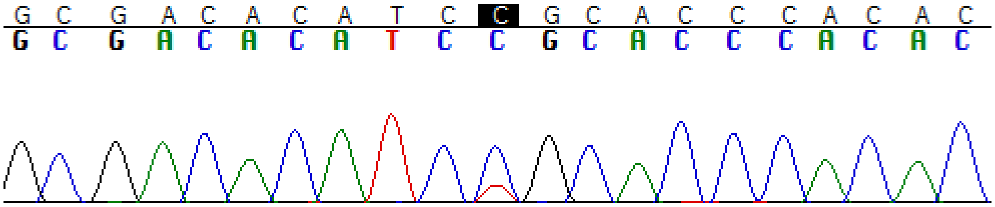

UPN#3-Diagnosis: NM_015898:exon2: c.522dupC:p.A175fs

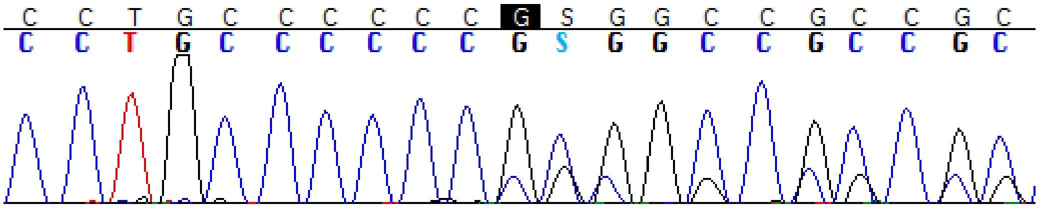

UPN#4-Diagnosis: NM_015898:exon2: c.149C>T:p.S50L

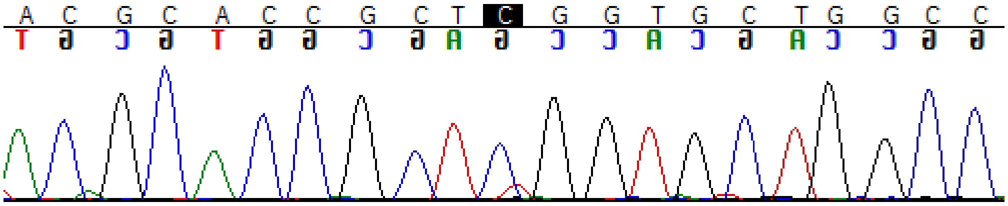

UPN#5-Diagnosis: NM_015898:exon2: c.1089_1090insTAA: p.V364delinsX

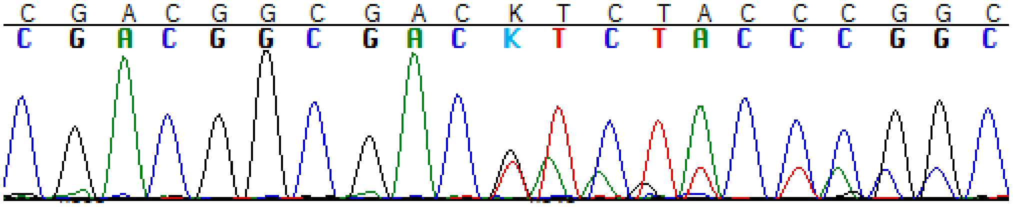

Supplementary Figure 1 (continued). Sanger sequencing confirms *ZBTB7A* mutations.

UPN#5-Diagnosis: NM_015898:exon2: c.146G>A:p.R49H

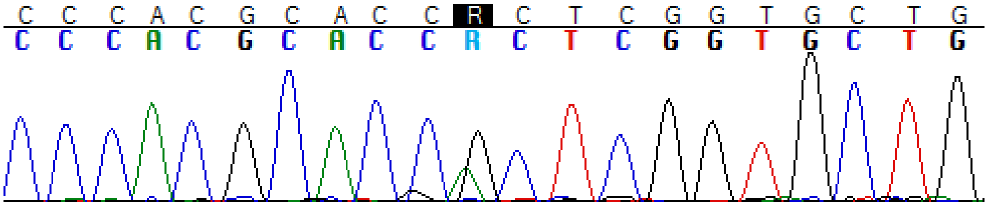

UPN#6-Diagnosis: NM_015898:exon2: c.1183G>T:p.G395C

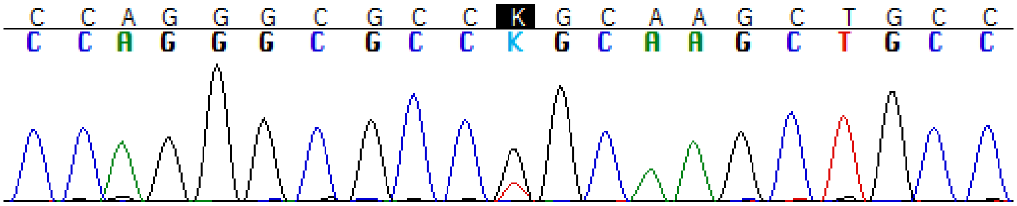

UPN#7-Diagnosis: NM_015898:exon2: c.1204C>T:p.R402C

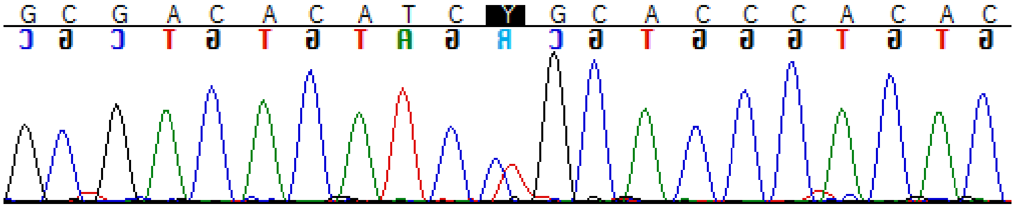

UPN#8-Diagnosis: NM_015898:exon2: c.522dupC:p.A175fs

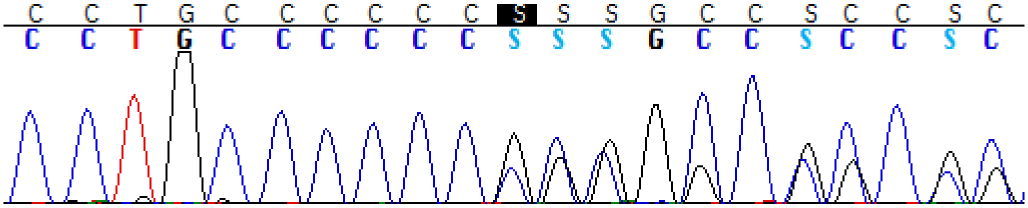

Supplementary Figure 1 (continued). Sanger sequencing confirms *ZBTB7A* mutations.

UPN#9-Diagnosis: NM_015898:exon2: c.1129C>T:p.R377X

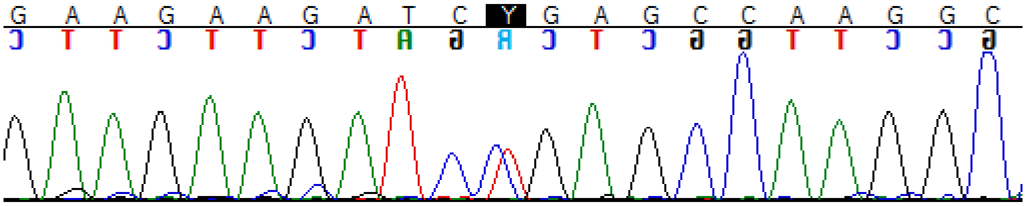

UPN#11-Diagnosis: NM_015898:exon2: c.237C>A:p.F79L

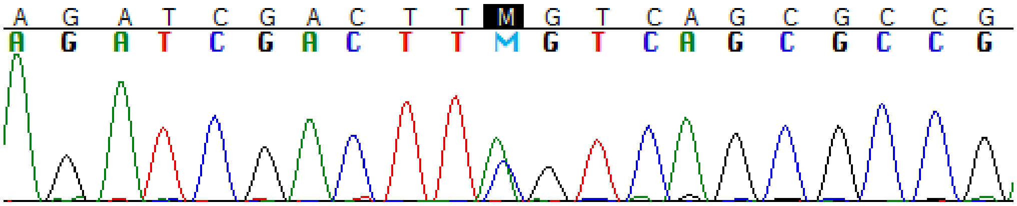

UPN#12-Diagnosis: NM_015898:exon2: c.522dupC:p.A175fs

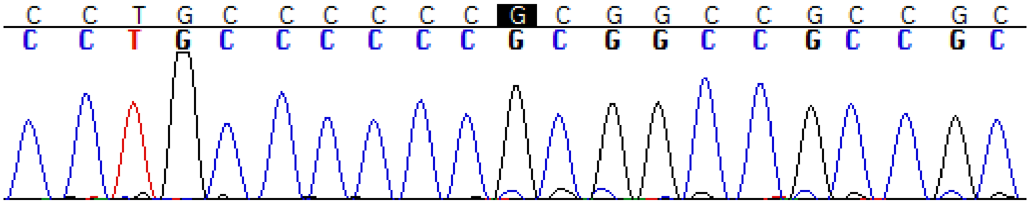

UPN#13-Diagnosis: NM_015898:exon2: c.254T>G:p.L85R

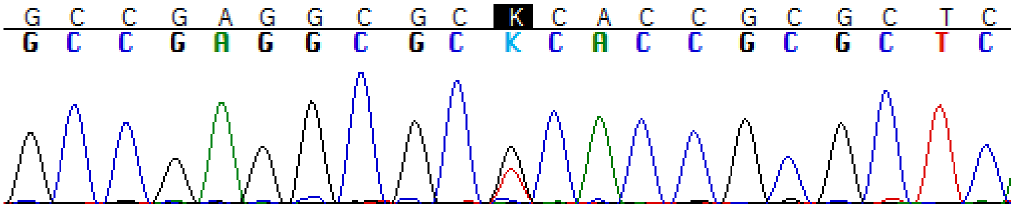

Supplementary Figure 1 (continued). Sanger sequencing confirms *ZBTB7A* mutations.

UPN#14-Diagnosis: NM_015898:exon2: c.522dupC:p.A175fs

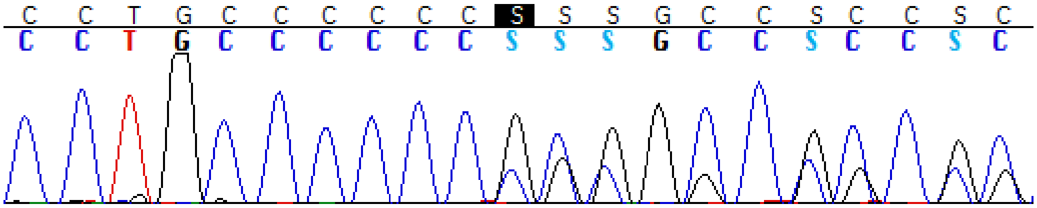

Supplementary Figure 1 (continued). Sanger sequencing confirms *ZBTB7A* mutations.

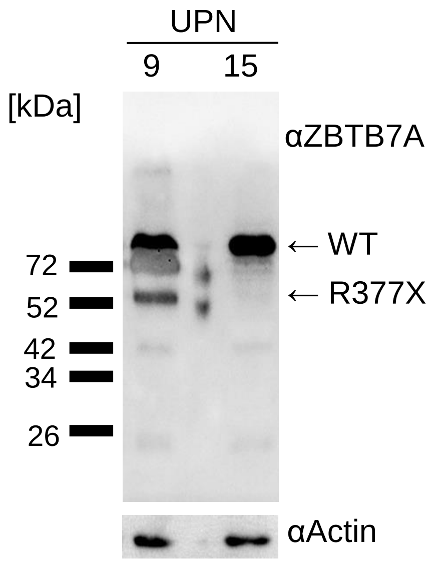

Supplementary Figure 2. Western blot analysis of a patient with the truncating R377X mutation (UPN9) and another patient with wild-type ZBTB7A (UPN15).

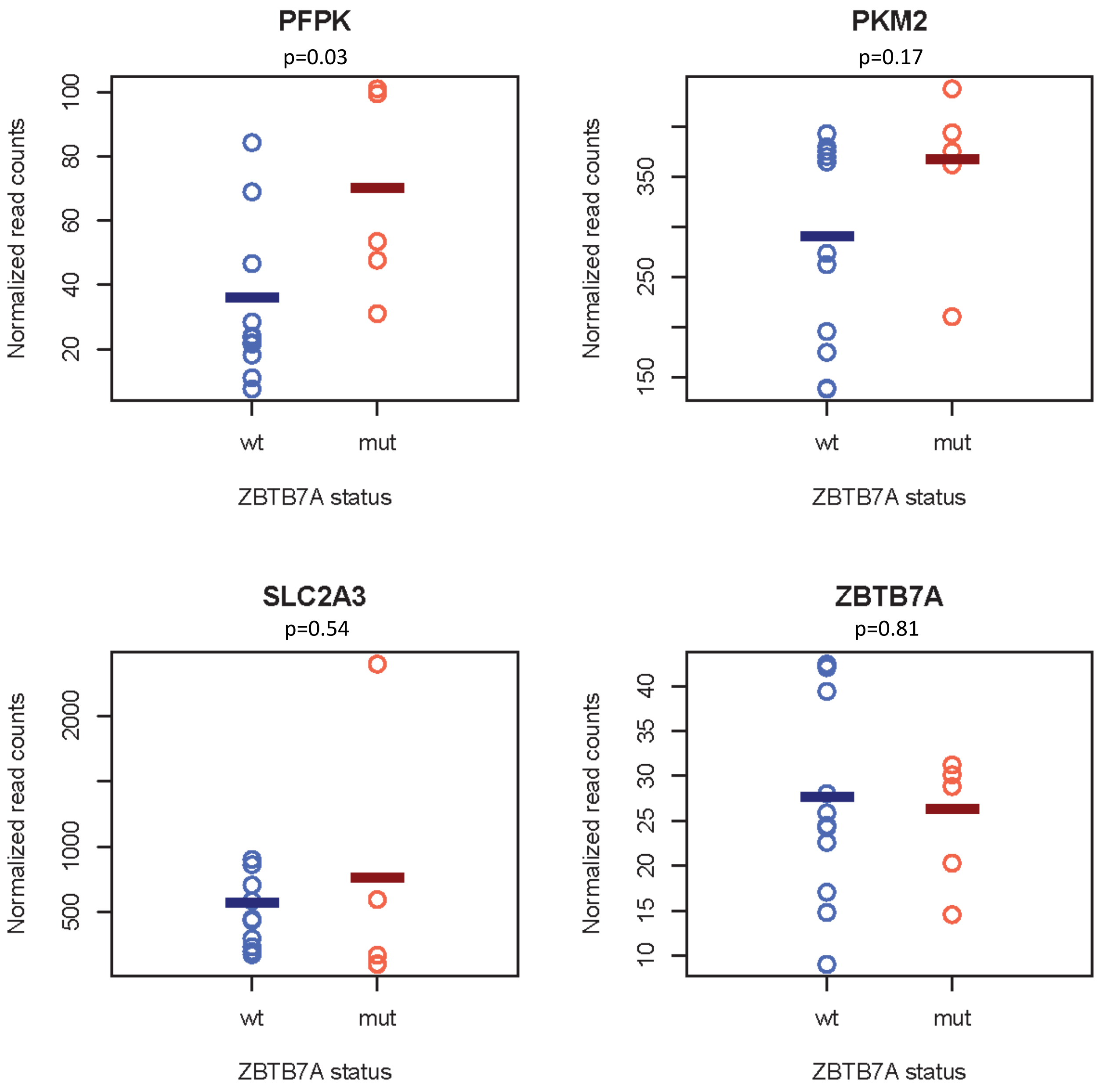

Supplementary Figure 3. Expression of glycolytic genes and *ZBTB7A* in AML t(8;21) patients according to *ZBTB7A* mutation status. Circles indicate mRNA sequence read counts from individual patients. Horizontal bars show mean values of the two patient groups (mutated n=5; wild-type n=11). Differences between groups were assessed using a two-tailed unpaired Student’s t-test.

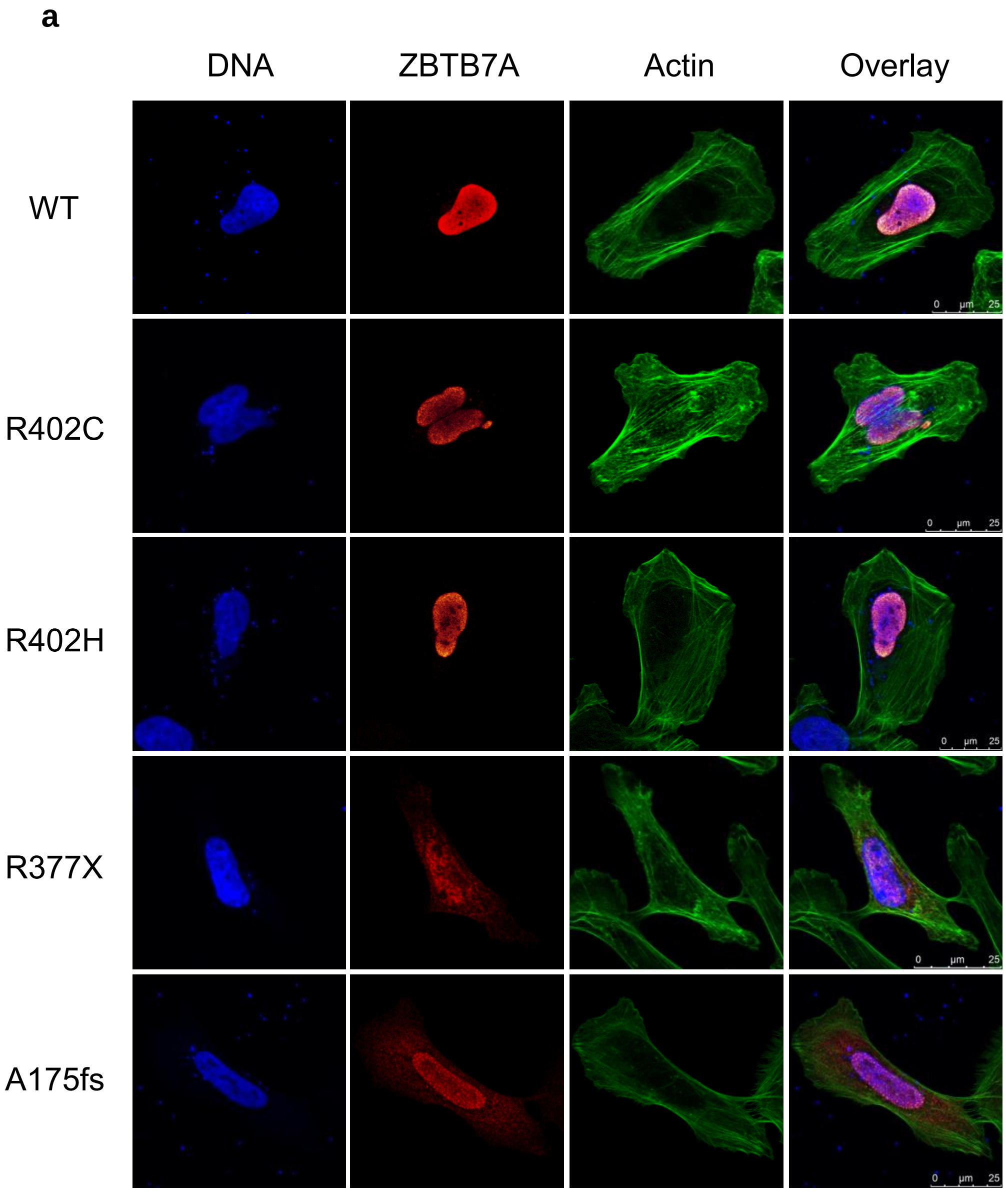

Supplementary Figure 4. Subcellular localization of ZBTB7A wild-type and mutants.
(a) Representative confocal laser scans of transiently transfected U2OS cells show the predominant protein distribution observed for each construct. Scale bar corresponds to 25 µm.

b

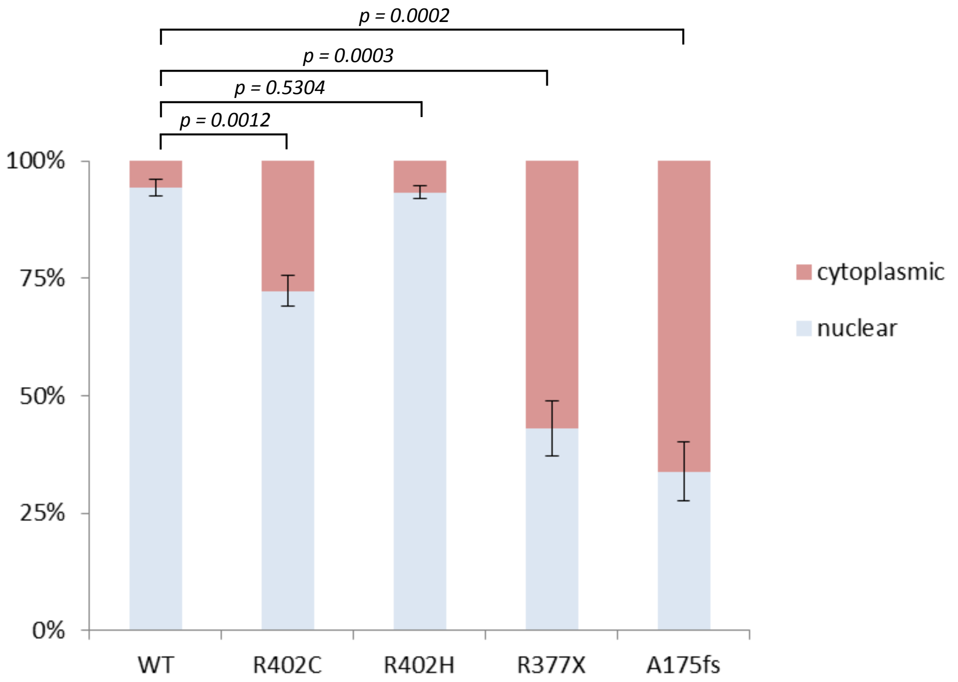

Supplementary Figure 4 (continued). (b) Cell counts after immunofluorescence staining of ZBTB7A wild-type and mutants in transiently transfected U2OS cells. Bar graph shows mean values ± standard deviation of 3 independent experiments with evaluation of 124 cells per construct (representing the minimum number of cells available for evaluation in each experiment). Statistical difference was assessed using a two-tailed unpaired Student's t-test. Nuclear localization was defined as detection of ZBTB7A exclusively in the cell nucleus, whereas cytoplasmic localization indicates ZBTB7A protein detected both in the nucleus and the cytoplasm.

c

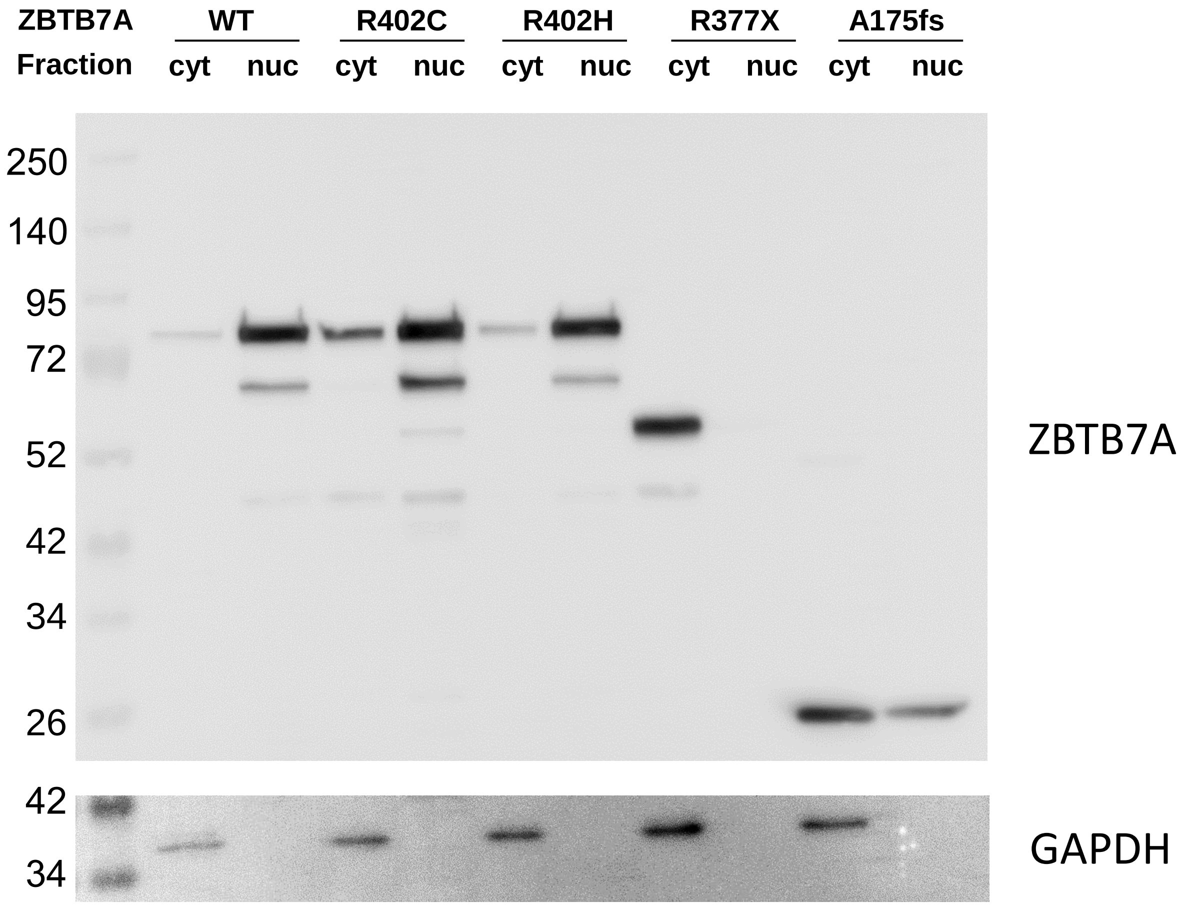

Supplementary Figure 4 (continued). (c) Western blot analysis of cytoplasmic (cyt) and nuclear (nuc) protein fractions extracted from HEK293T cells expressing ZBTB7A wild-type or mutants.

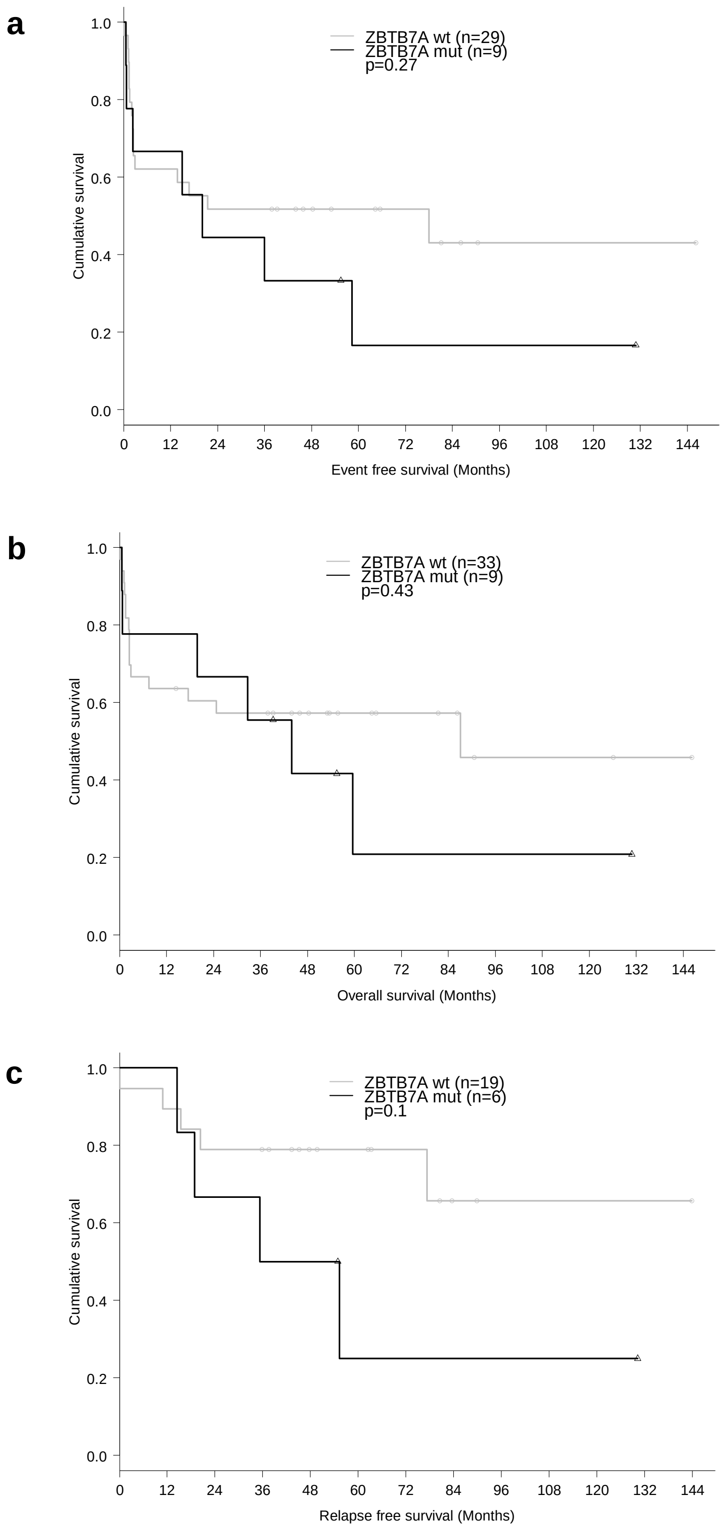

Supplementary Figure 5. Survival of t(8;21) positive AML patients according to *ZBTB7A* mutation status. P values were calculated by the log-rank test. (a) Event free survival, (b) Overall survival and (c) Relapse-free survival.

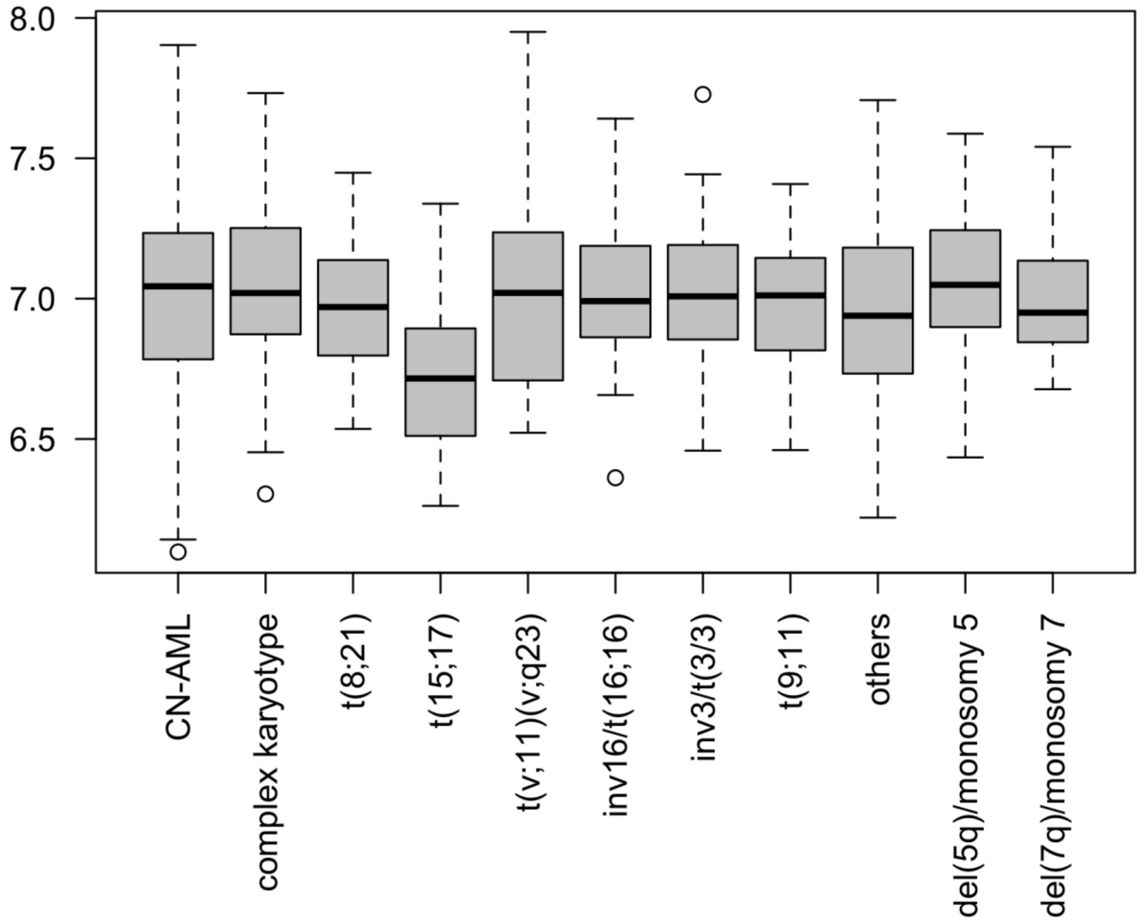

Supplementary Figure 6. *ZBTB7A* expression in cytogenetic subgroups of AML.

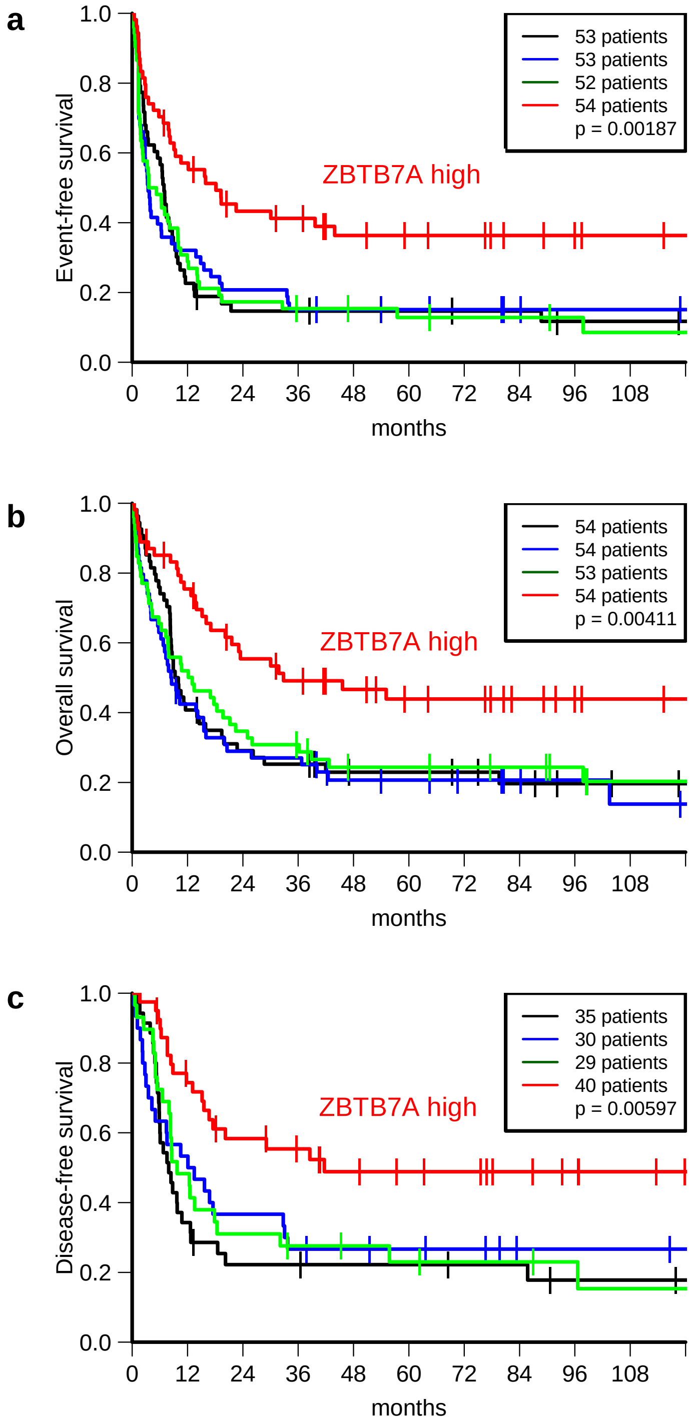

Supplementary Figure 7. Survival of patients with cytogenetically normal (CN-)AML according to *ZBTB7A* expression (GSE37642). High *ZBTB7A* expression (red) was defined as the highest (4th) quartile of expression values observed in CN-AML patients. Patients with *ZBTB7A* expression levels in the 1st to 3rd quartile were classified as having low expression. P values were calculated by the log-rank test. (a) Event-free survival (b) Overall survival (c) Relapse-free survival.

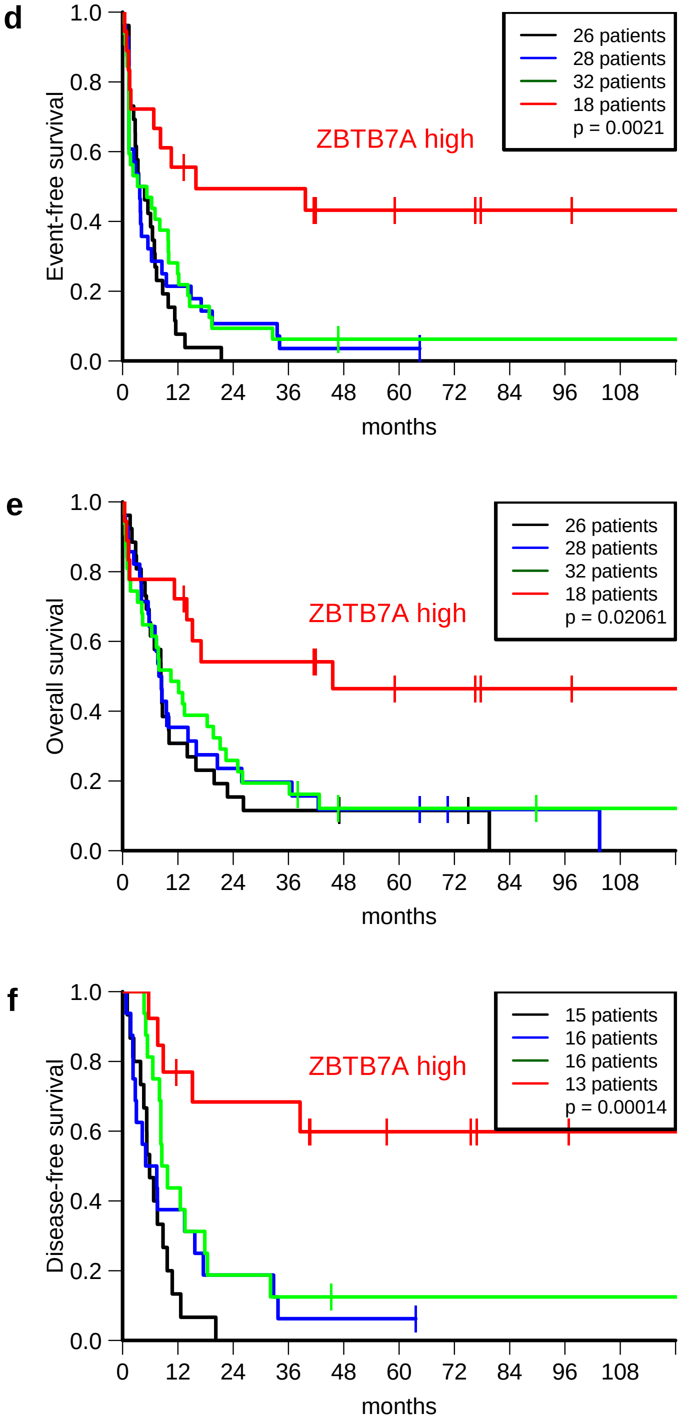

Supplementary Figure 7 (continued). Survival of **patients ≥ 60 years** with CN-AML according to *ZBTB7A* expression (GSE37642). (d) Event-free survival patients (e) Overall survival (f) Relapse-free survival.

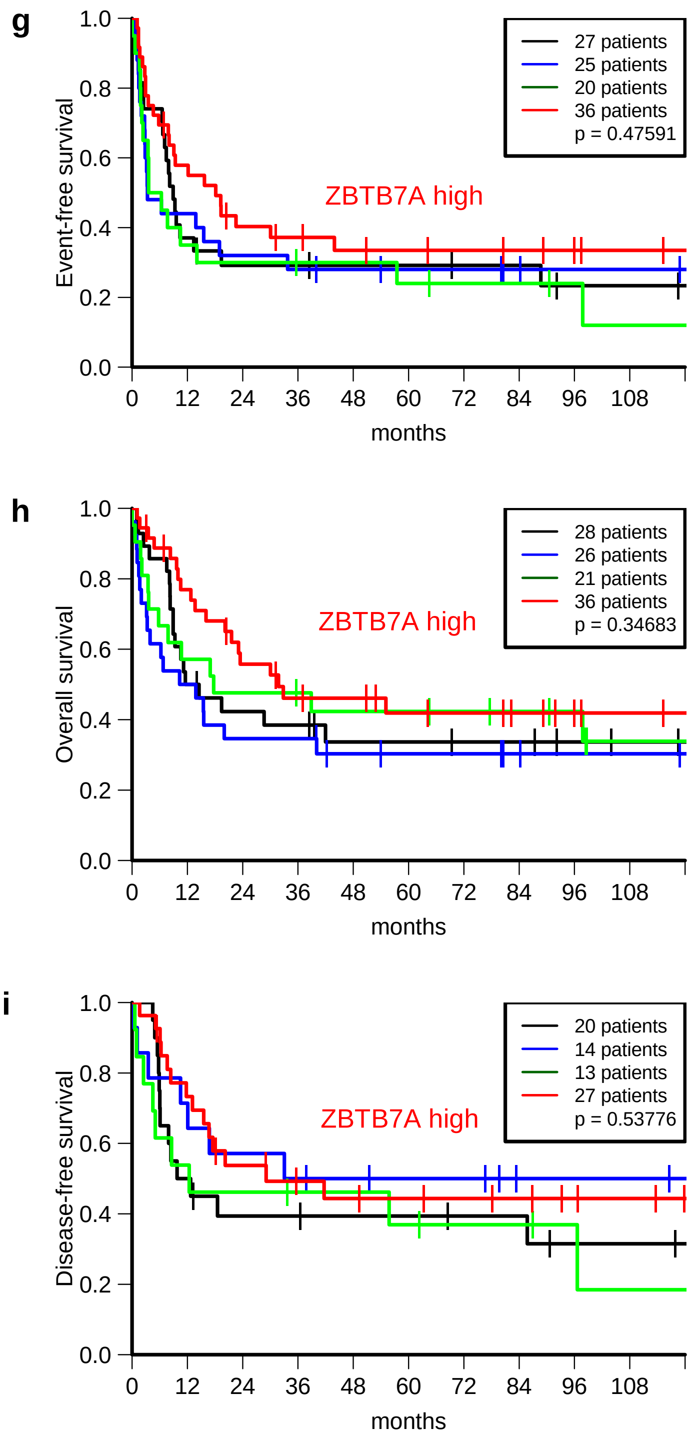

Supplementary Figure 7 (continued). Survival of **patients < 60 years** with CN-AML according to *ZBTB7A* expression (GSE37642). (g) Event-free survival patients (h) Overall survival (i) Relapse-free survival.

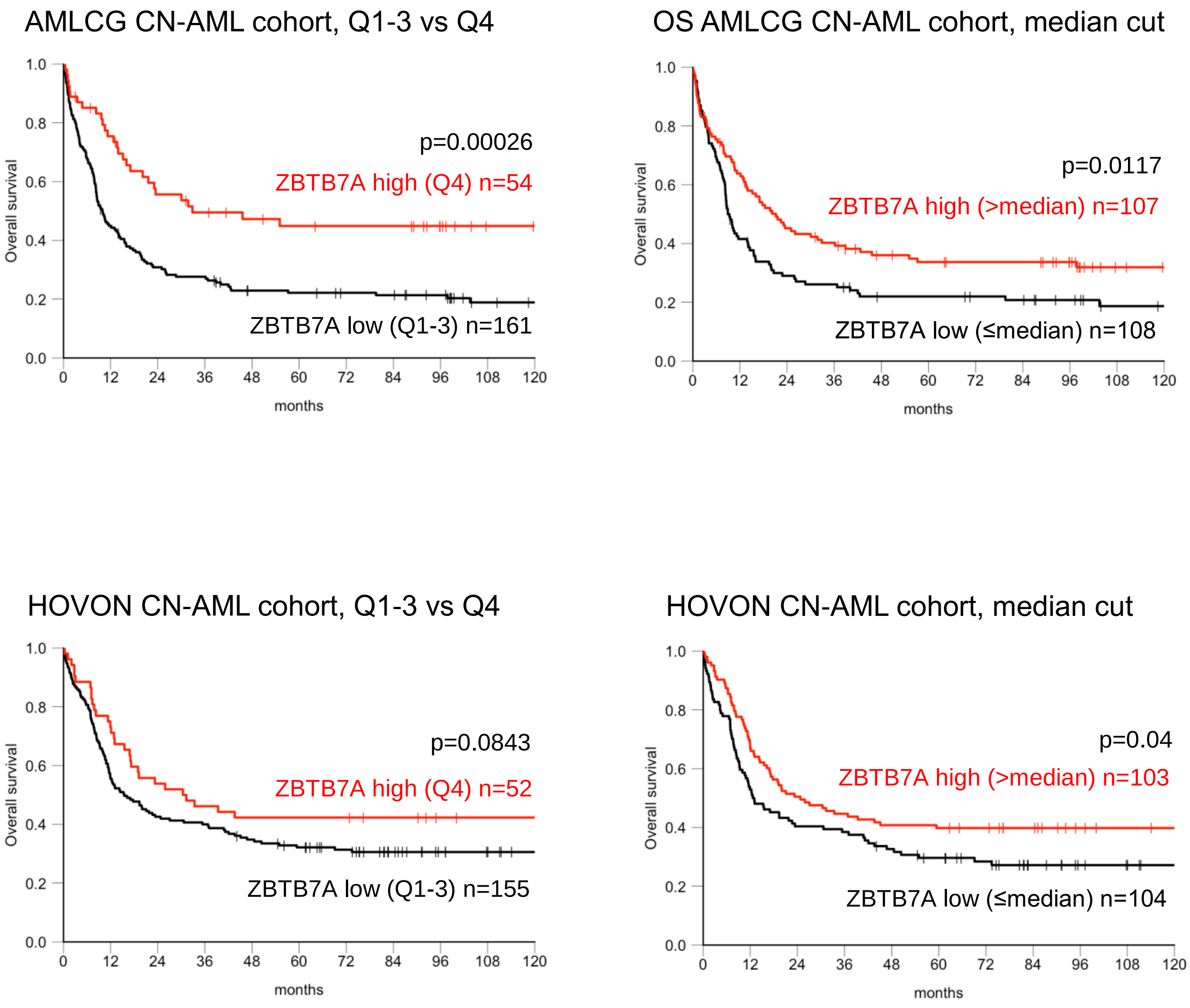

Supplementary Figure 8. Overall survival of patients with CN-AML according to *ZBTB7A* expression in the AMLCG-cohort (GSE37642) and the HOVON cohort (GSE14468 and GSE1159). P values were calculated by the log-rank test.

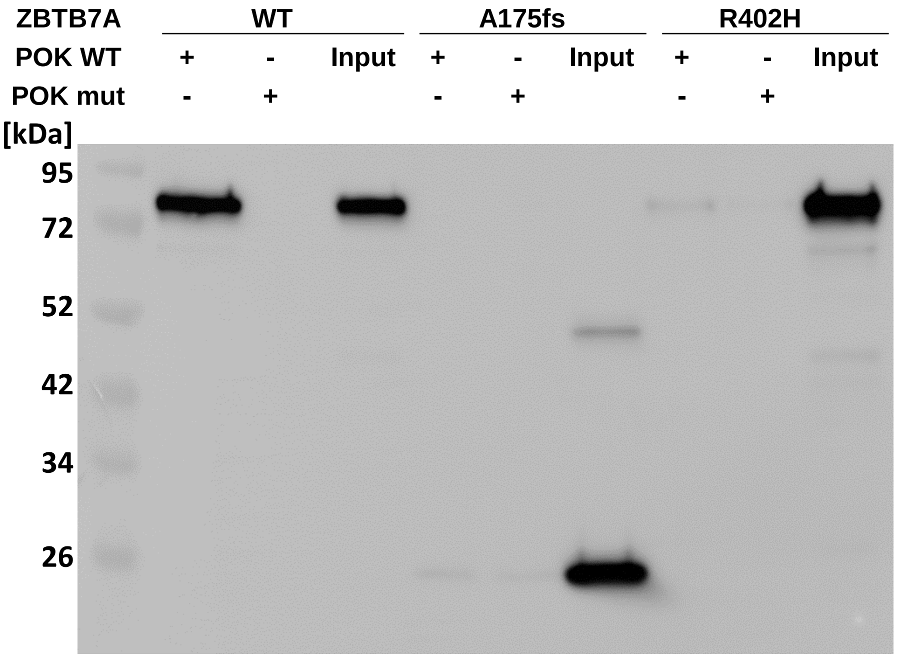

Supplementary Figure 9. Uncropped Western blot scan underlying Figure 2c.

Supplementary Table 1. Somatic variants from exome sequencing of two AML t(8;21) patients.

UPN	Chr	Position (hg 19)	Gene	Ref	Var	dbSNP	VarFreq (%)	Type	AA Change
1	7	138437432	ATP6V0A4	C	T		52.9	nonsynonymous SNV	NM_020632:c.967G>A:p.A323T
1	19	16040374	CYP4F11	T	A		87	nonsynonymous SNV	NM_021187:c.236A>T:p.Q79L
1	3	183823156	HTR3E	A	-GCAAG		50.5	frameshift deletion	NM_001256613:c.662_666delGCAAG: p.K222fs
1	5	38869211	OSMR	A	G		37.0	nonsynonymous SNV	NM_001168355:c.65A>G:p.Q22R
1	16	67695894	PARD6A	C	A		41.8	nonsynonymous SNV	NM_016948:c.385C>A:p.P129T
1	6	150569915	PPP1R14C	G	A		26.2	nonsynonymous SNV	NM_030949:c.457G>A:p.G153S
1	14	61186589	SIX4	G	A		45.1	stopgain SNV	NM_017420: c.1438C>T:p.Q480X
1	15	62994266	TLN2	C	G		47.8	nonsynonymous SNV	NM_015059:c.1772C>G:p.S591C
1	15	81627077	TMC3	C	G	rs376889456	35.4	nonsynonymous SNV	NM_001080532:c.2443G>C:p. E815Q
1	19	4054026	ZBTB7A	C	T		75.2	nonsynonymous SNV	NM_015898:c.1205G>A:p.R402H
1	19	24288837	ZNF254	G	A		43.9	nonsynonymous SNV	NM_203282:c.126G>A:p:M42I
2	12	70724077	CNOT2	C	T		44	nonsynonymous SNV	NM_014515:c.397C>T:p.P133S
2	1	22903142	EPHA8	C	T		34	nonsynonymous SNV	NM_020526:c.592C>T:p.R198C
2	16	30495266	ITGAL	C	T		29.7	nonsynonymous SNV	NM_002209:c.841C>T:p.R281C
2	4	55599321	KIT	A	T	rs121913507	28.3	nonsynonymous SNV	NM_000222:c.2447A>T:p.D816V
2	18	30321954	KLHL14	G	A		45.6	nonsynonymous SNV	NM_020805:c.1006C>T:p.R336W
2	5	140348603	PCDHAC2	G	T	position of rs143196630	37.2	nonsynonymous SNV	NM_018899:c.2252G>T:p.R751M
2	6	42890875	PTCRA	C	T		40.7	nonsynonymous SNV	NM_138296:c.169C>T:p.L57F
2	19	46198897	QPCTL	C	T		51.3	nonsynonymous SNV	NM_017659:c.554C>T:p.T185M
2	6	28540575	SCAND3	A	C		46.8	nonsynonymous SNV	NM_052923:c.3091T>G:p.S1031A
2	3	36534709	STAC	C	T		29.5	nonsynonymous SNV	NM_003149:c.754C>T:p. R252C
2	X	104464034	TEX13A	G	A		44.9	nonsynonymous SNV	NM_031274:c.842C>T:p.T281M
2	19	4054708	ZBTB7A	C	+G		45.7	frameshift insertion	NM_015898:c.522dupC:p.A175fs

Supplementary Table 2. Primer sequences for Sanger sequencing of *ZBTB7A* exon 2.

**Region**	**PCR-amplification primers**		**Sequencing primers**	
**Exon2_1**	fwd	GGGTGGAACGCTGCTTCT	fwd	CTTGTCAGTGGGCACAGGAA
	rev	GTTCATGGGGTTGCTCTGGA	rev	CTGAGGATGTCACCCACGTT
**Exon2_2**	fwd	GCTCATGGACTTCGCCTAC	fwd	ACAGCCAACGTGGGTGAC
	rev	GGTAGTAGTCCATGACGCCC	rev	CTCCCGACAGGAAGCCC
**Exon2_3**	fwd	CCAGAGCGGGATGAGGAC	fwd	ACTCTCCGGGCTTCCTGTC
	rev	GTGTGCACGTGCGTGTATG	rev	GTATGTGTGCGTCTGCGTG

Supplementary Table 3. *ZBTB7A* mutations from gene panel* analysis of 56 AML t(8;21) cases.

UPN	Chr	Position (hg 19)	Gene	Ref	Var	Length	Ref Count	Var Count	VarFreq (%)	Type	AA Change	Sanger validated
1	19	4054026	ZBTB7A	C	T	1	298	901	75.2	nonsynonymous SNV	NM_015898:exon2: c.1205G>A:p.R402H	Yes
3	19	4054027	ZBTB7A	G	A	1	564	136	19.4	nonsynonymous SNV	NM_015898:exon2: c.1204C>T:p.R402C	Yes
3	19	4054708	ZBTB7A	-	G	1	446	156	25.9	frameshift insertion	NM_015898:exon2: c.522dupC:p.A175fs	Yes
4	19	4054727	ZBTB7A	G	-	1	2387	290	10.8	frameshift deletion	NM_015898:exon2: c.504delC:p.P168fs	No
4	19	4055082	ZBTB7A	G	A	1	888	438	33.0	nonsynonymous SNV	NM_015898:exon2: c.149C>T:p.S50L	Yes
5	19	4054141	ZBTB7A	-	TTA	3	242	89	26.9	stopgain insertion	NM_015898:exon2: c.1089_1090insTAA: p.V364delinsX	Yes
5	19	4055085	ZBTB7A	C	T	1	305	211	40.9	nonsynonymous SNV	NM_015898:exon2: c.146G>A:p.R49H	Yes
6	19	4054048	ZBTB7A	C	A	1	522	77	12.9	nonsynonymous SNV	NM_015898:exon2: c.1183G>T:p.G395C	Yes
7	19	4054027	ZBTB7A	G	A	1	2129	1117	34.4	nonsynonymous SNV	NM_015898:exon2: c.1204C>T:p.R402C	Yes
8	19	4054708	ZBTB7A	-	G	1	459	231	33.4	frameshift insertion	NM_015898:exon2: c.522dupC:p.A175fs	Yes
9	19	4054102	ZBTB7A	G	A	1	235	167	41.5	stopgain SNV	NM_015898:exon2: c.1129C>T:p.R377X	Yes
10	19	4048131	ZBTB7A	G	-	1	328	35	9.6	frameshift deletion	NM_015898:exon3: c.1374delC:p.R458fs	No
10	19	4054708	ZBTB7A	-	G	1	208	12	5.5	frameshift insertion	NM_015898:exon2: c.522dupC:p.A175fs	No
11	19	4054994	ZBTB7A	G	T	1	462	174	27.4	nonsynonymous SNV	NM_015898:exon2: c.237C>A:p.F79L	Yes
12	19	4054708	ZBTB7A	-	G	1	7326	552	7.0	frameshift insertion	NM_015898:exon2: c.522dupC:p.A175fs	Yes
13	19	4054977	ZBTB7A	A	C	1	197	629	76.2	nonsynonymous SNV	NM_015898:exon2: c.254T>G:p.L85R	Yes
14	19	4054708	ZBTB7A	-	G	1	872	362	29.3	frameshift insertion	NM_015898:exon2: c.522dupC:p.A175fs	Yes

*JAK1, NRAS, GATA3, PTEN, SMC3, WT1, SF1, CBL, ETV6, KRAS, PTPN11, FLT3, IDH2, TP53, SRSF2, JAK3, CEBPA, U2AF2, DNMT3A, SF3B1, IDH1, ASXL1, RUNX1, U2AF1, SF3A1, MYD88, GATA2, KIT, TET2, FBXW7, IL7R, NPM1, BRAF, EZH2, RAD21, JAK2, NOTCH1, ZRSR2, BCOR, GATA1, SMC1A, STAG2, PHF6, ZBTB7A, ASXL2, FAT1

Supplementary Table 4. Patient characteristics of AML t(8;21) gene panel sequencing cohort.

Variable	Wild-type *ZBTB7A*	Mutated *ZBTB7A*	P value*
No. of patients	43	13	
Median Age, years (range)	55 (23-79)	53 (16-66)	0.148
Male gender, no. (%)	29 (67)	10 (77)	0.7331
White blood cell count G/l, median (range)	9 (1.9-210)	8.3 (3.5-245)	0.9689
Bone marrow blasts %, median (range)	70 (4-95)	55 (14-90)	0.1141
French-American-British (FAB) classification, no. (%)	M1: 7 (20)	M1: 1 (3)	0.6593
	M2: 28 (80)	M2: 10 (83)	1.0000
		M4: 1 (3)	0.2553
Secondary AML (%)	7	8	1.0000
Allogeneic transplantation, no. (%)	4 (12)	2 (22)	0.5928
Complete Remission, no. (%)	18 (55)	6 (67)	0.7083
Relapse, no. (%)	5 (28)	4 (67)	0.1501
Deceased, no. (%)	15 (45)	6 (67)	0.4537

*Two-tailed Fisher's exact test was used to compare categorical variables, while Wilcoxon Mann-Whitney U test was applied for continuous variables

Supplementary Table 5. *ZBTB7A* expression in molecular and age subgroups of CN-AML.

	All CN-AML<br>N=218			CN-AML <60 years<br>N=112			CN-AML >=60 years<br>N=106		
	$ZBTB7A^{Q4}$<br>N=55	$ZBTB7A^{Q1-3}$<br>N=163	P	$ZBTB7A^{Q4}$<br>N=37	$ZBTB7A^{Q1-3}$<br>N=75	P	$ZBTB7A^{Q4}$<br>N=18	$ZBTB7A^{Q1-3}$<br>N=88	P
FLT3-ITD	13/54	70/163	.015	11/36	36/75	.10	2/18	34/88	.03
NPM1	31/53	83/158	.52	20/36	47/74	.53	11/17	36/84	.11
LMR	24/53	34/159	.001	14/36	20/75	.20	10/17	14/84	<.001

ITD, Internal tandem duplication; LMR, low molecular risk genotype; mutated *NPM1* without *FLT3*-ITD, Q4, quartile of patients with highest expression levels of *ZBTB7A,* Q1-3, quartiles of patients with lower expression levels of *ZBTB7A.* P Values were calculated by two-tailed Fisher's exact test.
